# Fear of humans mediates spatiotemporal interactions between endangered large Asian felids

**DOI:** 10.1098/rspb.2026.1232

**Published:** 2026-10-07

**Authors:** Jiawei Feng, Dawei Wang, Zhilin Li, Xintong Wei, Jianping Ge, Tianming Wang

**Affiliations:** ^1^ Ministry of Education Key Laboratory for Biodiversity Science and Ecological Engineering, College of Life Sciences, Beijing Normal University, Beijing, 100875, People’s Republic of China; ^2^ National Forestry and Grassland Administration Key Laboratory for Conservation Ecology of Northeast Tiger and Leopard, Beijing, 100875, People’s Republic of China; ^3^ College of Life Sciences, Tianjin Normal University, Tianjin, 300387, People’s Republic of China

**Keywords:** *Panthera tigris*, *Panthera pardus*, competition, interspecies interaction, temporal activity pattern, camera trap

## Abstract

Species competitive interactions influence carnivore demography. Humans, as global ‘super predators,’ may mediate interactions among sympatric carnivores, although the effects of ecological fear on these interactions remain less understood. We used two years of camera trap data from more than 300 sites in northeast China to fit two-species, dynamic occupancy models. We then assessed how direct human passage affects spatial and temporal interactions between tigers and leopards, and the consequences for predator‒prey overlap in a temperate forest. We found evidence that median site occupancy, colonization and detection of leopards were lower at sites where tigers were present than at sites where tigers were absent. The effect of tigers on leopards in both space and time varied with disturbances. Leopards and tigers co-occurred less often than would be expected by chance at sites where human activity was greater. Tigers, but not leopards, significantly shifted their activity towards increased nocturnality in response to predominantly diurnal humans. This resulted in a decrease in temporal overlap among the two top predators and predator–ungulate prey at more disturbed sites, most notably between the tiger and prey (but no decrease in spatial overlap). Leopards appear to avoid tigers through time or space in areas where encounter risks are increased; in particular, leopards shift their core activity range as a main strategy to cope with dominant tiger and human activity across the sites. These results reveal that the human-induced landscape of fear may modify the strength of intraguild interactions between apex felids and structure food webs in a shared ecosystem. Furthermore, we highlight the importance of considering the pervasive risk introduced by a dynamic human presence to better understand the adaptive strategies used by competing top predators to mitigate threats.

## 1. Introduction

Large carnivores play pivotal roles in maintaining ecosystem functions and services through their top-down effects, and understanding their competition is essential for carnivore demography and conservation in the face of global carnivore declines [1–4]. Competitive interactions among carnivores within the same guild can shape species distributions, behaviour, population densities and community structure [5–7] and can arise when multiple species compete for similar space, time and/or food resources [5,8,9]. Sympatric carnivores typically coexist at various trophic levels within a system, with dominant predators having a top-down effect over subordinate species [10–12]. Apex carnivores may limit prey availability for subordinate species through interference or exploitative competition, forcing subordinate carnivores to forage under the threat of predation [7,8,13]. Additionally, the presence of dominant predators can force subordinate carnivores to alter their behaviour, such as shifting activity times or using less optimal habitats to avoid predation risk [12,14]. These behavioural adjustments are crucial for coexistence in a multi-predator/multi-prey system.

Intraguild predation among carnivores is a widespread ecological phenomenon [15,16] and can decrease the density and distribution of subordinate species through lethal and nonlethal interactions, leading to local exclusion or extirpation [10,17,18]. Carnivores under such conditions face a trade-off between risk avoidance and resource acquisition to reduce fatal encounters with dominant competitors while increasing their ecological benefits by partitioning their niches along spatial, temporal and/or dietary axes [19–21]. Studies in tropical forests of India have shown that tigers (*Panthera tigris*), leopards (*P. pardus*) and dholes (*Cuon alpinus*) vary in their use of space and time to facilitate coexistence, with fine-scale avoidance behaviours especially apparent in areas of high carnivore and prey densities [21].

Humans and other animals are increasingly sharing the world's landscapes, even in protected areas, and interacting at an unprecedented scale [22,23]. Animals worldwide must increasingly contend with novel risk landscapes introduced by humans. Fear of humans plays an important role in mediating spatiotemporal interactions among sympatric carnivores, determining how these species coexist and use space and time in multiuse landscapes [24–26]. Large carnivores perceive humans as ‘super predators’, which induces intense fear responses from animals, changing their habitat use, activity patterns, movement and foraging behaviour [27–30]. Fear leads carnivores to adjust their temporal activity patterns, often becoming more nocturnal to avoid predominantly diurnal humans. Such temporal shifts reduce the availability of temporal refuges for prey, altering predator‒prey encounter rates and dynamics [31–33]. In multiple carnivore systems, these behavioural adaptations to human presence affect not only predator‒prey interactions but also interspecific interactions among carnivores. For example, tigers, leopards and dholes in Bhutan may exhibit spatial and temporal segregation to reduce competition and avoid both humans and dominant carnivores [24]. Understanding how humans can mediate spatiotemporal interactions among carnivores is timely and warranted as the world becomes increasingly crowded.

Tigers and leopards are among the most iconic carnivores in Asia, and they are well known for their influence on ecosystem functions such as carbon storage and trophic level balance [34,35]. Interactions among tigers, leopards and humans involve competition, predation and behavioural adaptations at different spatiotemporal scales. Tigers, as dominant apex predators, influence the behaviour of leopards, causing the latter to employ differential spatial and/or temporal avoidance strategies and diet partitioning to avoid competitive encounters [4,18,21]. For example, in northeast Asia, tigers are mostly nocturnal, whereas leopards are more diurnal, implying temporal partitioning as a coexistence strategy [20]. In Nepal, tigers spatially overlap with people on foot and vehicles while offsetting their temporal activity patterns to be much less active during the day when human activity peaks [36].

Growing human pressures in Asia are likely to further intensify tiger and leopard responses to humans, as well as affect their spatial or temporal overlap with ungulate prey; these interactions are crucial for the structure and functioning of food webs in Asian forests. Tigers and leopards tend to segregate spatially in areas with high human pressure, with leopards moving closer to human settlements, increasing human‒wildlife conflict risk and retaliation [24,37]. Although wide-ranging tigers and leopards co-occur, it is not well understood whether anthropogenic threats, specifically the temporal dynamic of human presence at the site level, mediate their spatiotemporal interactions. Understanding the behavioural mechanisms that allow two large felids to coexist in areas with human activity is key to providing insights into the drivers of community structure across space and time, and improving the conservation of large carnivore populations in shared, complex landscapes [20,24].

Here, we quantified how direct human activity captured on camera traps may reshape behaviour and spatiotemporal interactions between tigers and leopards, as well as their consequences for spatial and temporal predator‒prey overlap via two years of camera trap data from sites distributed across northeast China. We used Bayesian two-species occupancy models to assess the evidence for an effect of tiger presence on site occupancy, colonization and persistence by leopards in relation to human presence. We used multinomial logit mixed models and piecewise exponential additive mixed models to assess changes in the spatial and temporal activity of tigers and leopards across the observed range of human activities. We also used the coefficient of activity pattern overlap from the kernel density estimates to test for changes in two predator and predator‒prey pairs at great and low human sites. Because tigers competitively suppress subordinate carnivores [4,18], we expected leopards to avoid competitive interactions by adjusting their spatiotemporal activity patterns when tigers are present, which also depends on how both species respond to site-level human risk. We expected that as the intensity of human activity increases, large carnivores reduce the risk of potentially fatal encounters by shifting their activity towards increased nocturnality to avoid predominantly diurnal humans, resulting in a decrease in the diel overlap with their major ungulate prey. We also expected that large carnivores reduce spatial overlap with their major prey species at high human sites, because predators and prey may differ in their spatial responses to human disturbance.

## 2. Methods

### (a) Study area

Our study area was located in the eastern part of Northeast Tiger and Leopard National Park (NTLNP), China (supplementary material, figure S1). The national park covers an area of 14 100 km^2^ and encompasses the core areas occupied by 89% of all Amur tiger (*P. t. altaica*) and Amur leopard (*P. p. orientalis*) individuals in China, including the only stable breeding habitat of these large felids in China [38,39]. Early studies suggested that tiger and leopard densities are relatively low compared to those in India and Nepal (0.20–0.27 adult tigers and 0.30–0.42 adult leopards 100 km^−2^, respectively) in the area [40,41]. Their primary prey includes wild boar (*Sus scrofa*), sika deer (*Cervus nippon*) and Siberian roe deer (*Capreolus pygargus*), contributing over 80% and 75% of the dietary biomass for tigers and leopards, respectively [42,43]. Human activities in the forest are pervasive (figure 1). For more detailed information on the study area, please refer to Feng *et al.* [44].

**Figure 1.**
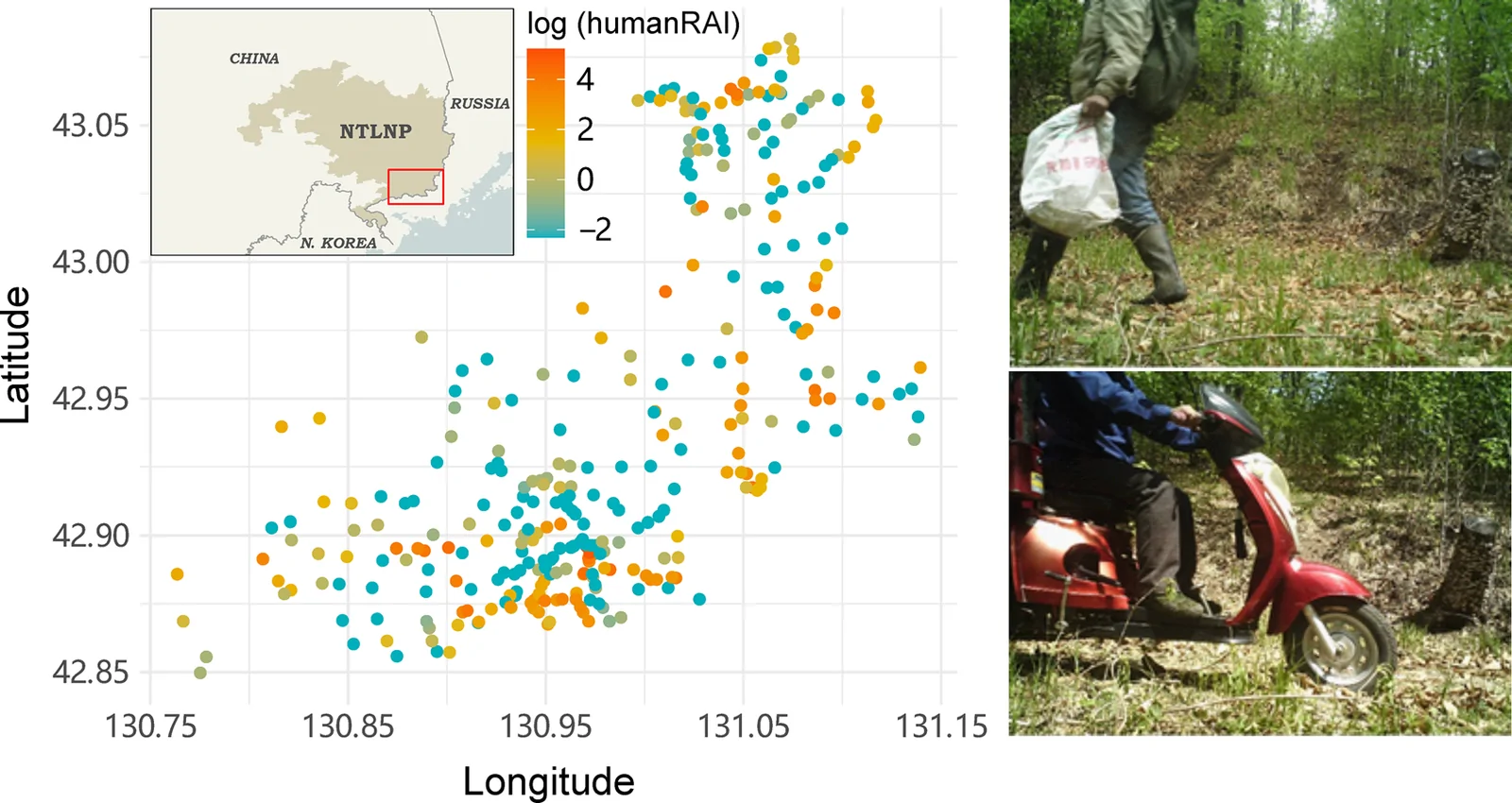
Map of our camera-trapping study area in northeast China monitored in 2018 and 2019. Sampling locations with different colours indicate different human disturbance intensities estimated using the camera-trap-derived relative abundance index (RAI) of human activities (people and vehicles). Photographs on the right show human presence (i.e. people on foot and vehicles) in the study area. Photo credit: T. Wang. The insert shows the location of the study area along the China–Russia border.

### (b) Camera trap surveys

From January 2018 to September 2019, we deployed infrared motion-sensor cameras to collect field data on tigers, leopards, their main prey species (sika deer, roe deer and wild boar), and human presence (local residents, army personnel and vehicles) at each site (supplementary material, figure S1). We sampled 107 sites in 2018 (1 January–31 December for 70 sites and 18 June–31 December for 37 sites) and 322 sites in 2019 (14 January–30 September), for a total of 329 unique sites. The average distance between adjacent camera stations was 519 m (s.d. = 374 m). We maximized the detection probability by placing cameras at sites where mammals were likely to travel (e.g. along ridges, trails and forest roads). A single unbaited camera was positioned approximately 60 cm above the ground and was operated 24 h day^−1^ with 1-min quiet intervals between consecutive 10 s videos. We excluded false triggers and research teams from the analyses. We defined independent detection events for each species using a 30 min quiet period between consecutive detections in the ‘camtrapR’ package in R [45], regardless of the number of species in the video.

### (c) Spatial analysis

All the statistical analyses were conducted in R version 4.4.0 [46]. Using detection data from 2019 (February–September), we quantified whether tiger presence influenced leopard site use by applying a Bayesian dynamic occupancy framework designed for interacting species, following approaches by Gallo *et al.* [47] and Richmond *et al.* [48]. We tagged site- and time-stamped videos for the detection (1) or non-detection (0) of leopards and tigers each month. We removed camera sites for which there were fewer than 60 working days, retaining a total of 311 sites. A site was treated as occupied by a species if at least one image of that species was captured within a given month. The modelling approach assumes that the occupancy dynamics of the subordinate species (leopard) can be influenced by the presence of a dominant predator (tiger), reflecting hierarchical interactions across trophic levels. For tigers, we estimated four key parameters across sites (*i* = 1,…, *I*) and sampling periods (*t* = 1,…, *T*). To simplify notation, site and season subscripts are omitted here. These parameters included initial occupancy at *t* = 1 (ψ*^t^*), colonization (γ*^t^*), persistence (ϕ*^t^*) and detection probability (ρ*^t^*). Leopard dynamics were formulated as conditional on tiger presence. Accordingly, we estimated eight parameters: the probability of initial leopard occupancy when tigers were present or absent (ψ*^l|t^*, ψ*^l|t^^-^*), the probability of colonization (γ*^l|t^*, γ*^l|t^^-^*), the probability of persistence (ϕ*^l|t^*, ϕ*^l|t^^-^*) and detection probability given tiger presence or absence (ρ*^l|t^*, ρ*^l|t^^-^*). We modelled the eleven parameters using a logit link function and incorporating site-level covariates. We recognize that leopard–tiger interactions are shaped by numerous biotic and abiotic factors, including elevation, vegetation structure and prey availability [40,44]. However, on the basis of early leopard research conducted within this park [49], we centred our analysis on direct human presence as the main factor linked to reduced leopard occupancy. Therefore, human disturbance was included as a predictor in all logit-linear components, including initial occupancy, colonization, persistence and detection, to evaluate whether tiger and leopard interactions spatially varied across the forest landscape. Human impact was quantified using a human relative abundance index (human RAI), defined as the number of independent human detections per 100 camera trap days at each camera site over the study period. To address the highly right-skewed distribution and improve model convergence, human RAI were transformed using log (*x* + 0.1) before analysis.

We quantified whether tigers and leopards co-occurred more or less than expected by chance by calculating species interaction factors (SIFs) along the gradient of human disturbance from the model-derived probabilities [12,47,48]. An estimated SIF less than 1 indicates that two species co-occurred less frequently than expected by chance, and an SIF more than 1 indicates that a species co-occurred more frequently than expected by chance.

We followed the approach of Gallo *et al.* [47] and Richmond *et al.* [48] to calculate an SIF such that SIF = ψ*^t^*ψ*^l|t^*/ψ*^t^* (ψ*^t^*ψ*^l|t^* + (1 − ψ*^t^*) ψ*^l|t^^-^*), where ψ*^t^* is the unconditional occupancy probability of a tiger, ψ*^l|t^* is the occupancy probability for leopards given that tigers are present, and ψ*^l|t^^-^* is the conditional probability of leopard occupancy when tigers are absent. Since occupancy at any given time depends on both colonization and persistence, we employed the recursive formulation of [50] recursive equation of MacKenzie *et al.* [50], ψ*_t_* _+ 1_ = ψ*_t_*ϕ*_t_* + (1 − ψ*_t_*)γ*_t_*, to estimate subsequent occupancy probabilities in each survey period. These values were then used to calculate SIFs for tiger-leopard interactions across sampling seasons [12].

We fitted this model within a Bayesian framework implemented in JAGS (v. 4.3.1) using the ‘runjags’ package [51]. We ran 12 chains to sample the posterior distribution 200 000 times while following a 10 000-step adaptation and 10 000-step burn-in phase. The chains were thinned by two for a total of 100 008 posterior samples. Model convergence was assessed by visually inspecting trace plots and ensuring that the Gelman‒Rubin diagnostics were less than 1.1 [52]. All the parameters in our model achieved convergence. For parameters on the logit scale, we reported 95% credible intervals (CIs) and considered an effect present when the CI did not overlap zero. For SIF values, we used the same approach but checked whether the CI overlapped with 1.

Notably, because of the small detection area of cameras relative to species movement and home range size, camera data often violate the occupancy model assumptions of site closure and spatial independence [53]. The home ranges of tigers and leopards exceed 100 km², and those of ungulates exceed 1 km² [20,40], potentially creating detection correlations between nearby sites. We therefore treated movement as random and interpreted occupancy as the probability of use***—***the asymptotic likelihood of a site being used over the eight-month sampling period [50,53].

Finally, we compared the spatial overlap between each apex predator (tigers and leopards) and their sympatric ungulate prey (sika deer, roe deer and wild boar) using species abundance data between sites of low and high human use. Habitat use by leopards was the primary influence on tiger presence at sites with high human RAIs (more than 4.11), where co-occurrence was less common (SIF less than 1 and SIF CI did not overlap 1; see *Results*). Thus, we defined sites with low versus high human use using a threshold of human RAI = 4.11. We calculated the relative abundance index (RAI) for each species at each trap site as the number of detections per 100 camera-trap days. Each camera trap was considered an independent spatial point, and Pianka's index [54] was estimated using RAI to reflect the predator–prey spatial overlap across human-use categories. The indices were calculated with the function *niche.overlap* in the *spaa* package [55].

### (d) Temporal analysis

To assess how human passage affects the presence of tigers or leopards at the scale of hours to days (i.e. reactive temporal interactions), we used recurrent event analysis and the piecewise exponential additive mixed model (PAMM) [56] to estimate the nonlinear temporal dynamics of the visitation rate of a predator after a human presence. Based on the procedure outlined by Ferry *et al.* [57], we estimated predator hazard rates, defined as the distribution of waiting times between consecutive detections of a species following human presence. The survey window was defined as a 25-day time period after any human presence. To address repeated observations at the same location, the camera site was included as a random effect. Analyses were performed in R using the packages *ctrecurrent* [57] and *pammtools* [56]. Furthermore, to test whether the nocturnal behaviour of two predators and major prey differed in response to human presence, we fitted multinomial logit mixed models (MNLMMs) using two variable categories (i.e. day and night) as the response variable. These models tested whether the probability of detection in each diel period varied with disturbance [58]. The camera site and month were included as random effects in all the models, and restricted maximum likelihood (REML) was applied. We included month-level random effects to statistically control for temporal variation in detection probabilities (e.g. due to weather or other unmeasured seasonal factors). The day period was used as the reference category. We categorized camera trap videos based on the time of day two predators were captured. Nighttime videos were defined as those occurring between sunset and sunrise, while daytime videos were defined as those occurring between sunrise and sunset.

Then, we considered the intensity of human activity as a site-level factor that could mediate temporal partitioning between leopards and tigers. Similar to spatial analysis, we defined sites with low versus high human use using a threshold of human RAI = 4.11. For both tigers and leopards, diel activity patterns were characterized with circular kernel density estimation, and 50% isopleths (activity cores) were obtained with the function *modal.region.circular* from the *circular* R package [59], representing the realized temporal niche of each predator under differing human-use conditions. Finally, we compared the temporal overlap between each apex predator (tigers and leopards) and their ungulate prey from circular activity distributions between sites of low and high human use. Overlap coefficients (Δ) were calculated with the function *OverlapEST* in the *overlap* package [60]. The coefficient Δ represents the proportion of overlap between two activity distributions, with values ranging from 0 (no overlap) to 1 (complete overlap) [61]. Following Ridout and Linkie [61], we chose to use the Δ_4_ nonparametric estimator to arrive at our overlap coefficient recommended for large sample sizes (*n* > 75 events). To quantify uncertainty, we generated 95% confidence intervals (CIs) for Δ using 10 000 bootstrap replicates for each species pair. Differences in predator–predator and predator–prey overlap across human-use categories were assessed by comparing CIs of Δ. The function *compareCkern* in the *activity* R package [62] was used to test for significant differences (*p* < 0.05) in temporal activity among species. To standardize activity patterns, we converted clock time to solar time using the sunTime function in the overlap package, setting 06.00 as sunrise and 18.00 as sunset. Owing to the limited detection of predator species, observations from both survey years were pooled into a single dataset for analysis.

## 3. Results

Across 78 795 trap nights, we recorded 971 independent detections of Amur tigers and 455 of Amur leopards. These detections included at least 12 individually identified tigers (five adult males, six adult females and one unidentified adult) and 26 individually identified leopards (12 adult males and 14 adult females) within the study area from January 2018 to September 2019. We also recorded 20 584 independent detections of ungulates (sika deer = 15 118, roe deer = 2083 and wild boar = 3383) and 7160 independent detections of humans, including vehicles (supplementary material, table S1).

### (a) Multispecies occupancy

Detection-corrected estimates showed that the median initial occupancy of tigers was 0.239 (95% CI = 0.134–0.388; supplementary material, table S2). Site colonization by tigers was 0.133 (95% CI  =  0.096–0.185), and persistence was 0.480 (95% CI  =  0.277–0.682; supplementary material, table S2). Humans were positively related to both colonization (0.34, 95% CI = 0–0.67) and persistence (1.06, 95% CI = 0.38–1.74), suggesting that tiger occupancy dynamics were strongly shaped by variation in human activity.

For the leopards, the detection and colonization rates were significantly lower at the sites where tigers were present than at the sites without tigers. Moreover, in these tiger-occupied areas, human passage substantially lowered the chances of leopard colonization (−2.58, 95% CI = −5.74 to −0.98) and detection (−3.42, 95% CI = −3.78 to −3.08; supplementary material, tables S3 and S4). This finding indicates that leopards were less likely to colonize and harder to detect in areas with greater human activity when tigers were present.

Furthermore, leopards showed a higher probability of initial occupancy at sites without tigers (0.429, 95% CI = 0.297–0.581) compared with those where tigers occurred (0.024, 95% CI = 0.013–0.044; table 1). A complete set of parameter estimates with corresponding interpretations is provided in the supplementary material (tables S3 and S4).

### (b) Spatial behaviour

The SIF between tigers and leopards decreased as the human RAI increased. Specifically, at sites where the human RAI was more than 4.11 (log[humanRAI] = 1.44), the SIF tended to be less than 1, and the 95% CI did not overlap 1, indicating sufficient evidence to determine leopard spatial avoidance towards tigers (figure 2). SIFs were more than 1 between tigers and leopards at sites where the human RAI ranged from 0 to 4.11, but the 95% CI overlapped with SIF  =  1 (figure 2; see supplementary material, figure S2 for SIF by month); thus, leopards were not spatially associated with tigers in areas with lower levels of human disturbance (supplementary material, figure S3).

**Figure 2.**
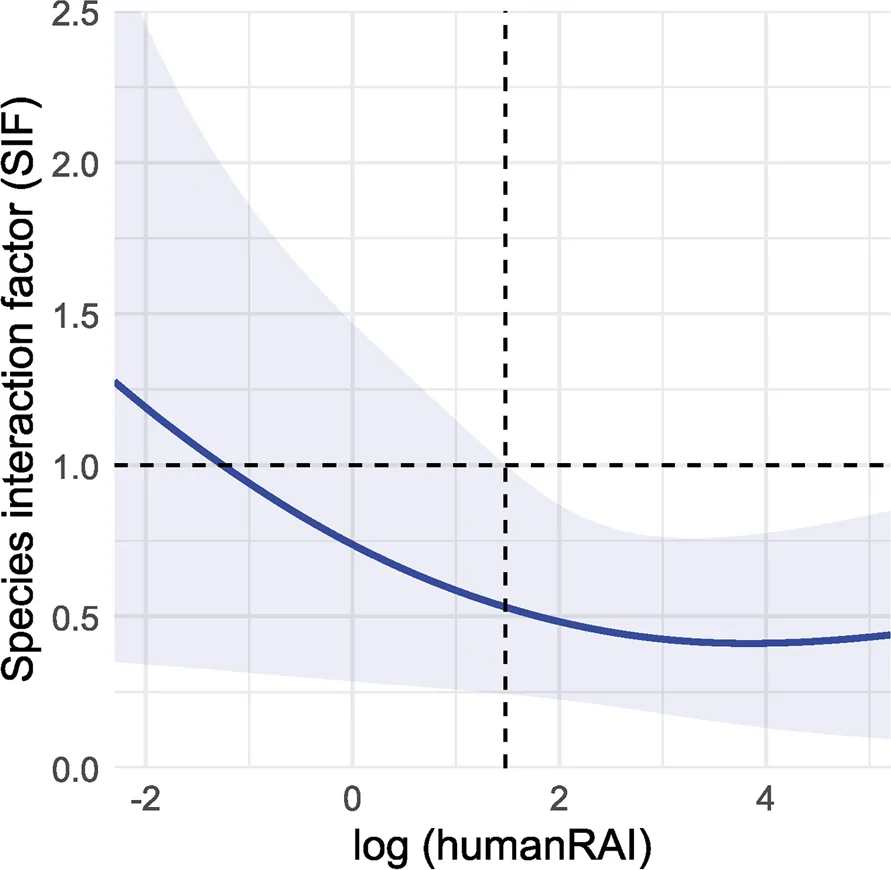
Species interaction factor (SIF) of leopards and tigers in relation to human activity in northeast China. An SIF of 1 indicates expected spatial overlap, whereas an SIF more than 1 indicates that the species interact more often than expected, and an SIF less than 1 indicates that the species interact less often than expected. The shaded areas show the 95% confidence intervals. The vertical dotted line represents log[humanRAI] = 1.44.

Furthermore, disturbance altered the spatial overlap between predator and prey (figure 3). Tiger–prey overlap was consistently higher at more disturbed sites than at less disturbed sites, especially for sika deer (Pianka's index: 0.58 [0.49–0.67] vs 0.37 [0.28–0.47]; 95% CIs did not overlap) and roe deer (0.46 [0.36–0.56] vs 0.27 [0.18–0.39]; figure 3a). By contrast, human activity did not significantly alter spatial overlap between leopards and three prey species (figure 3b).

**Figure 3.**
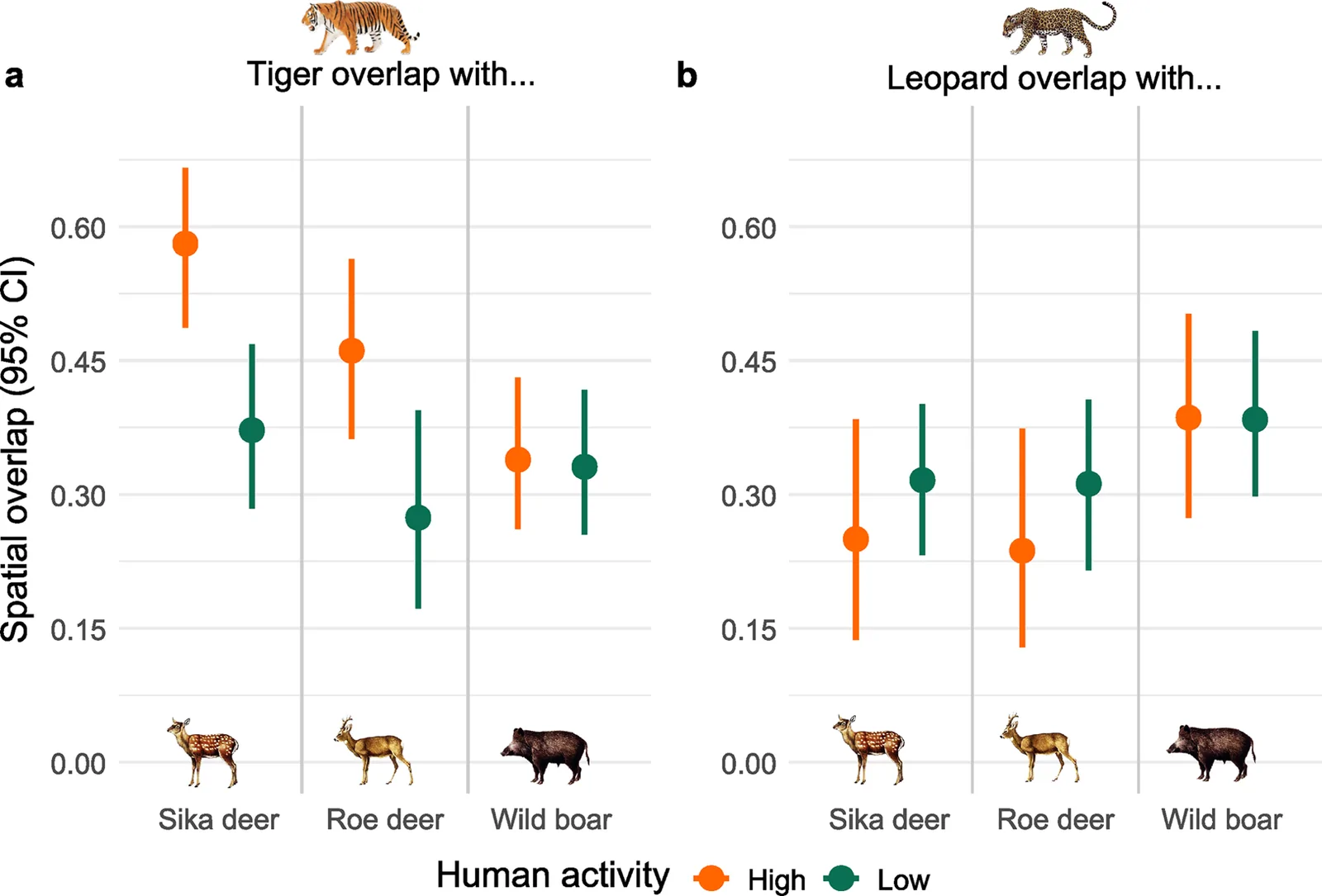
Changes in spatial overlap (Pianka's index) of (a) tigers and (b) leopards with their major prey species between sites of low and high human activity. The index value ranges from 0 (no overlap) to 1 (complete overlap). The error bars show the bootstrapped 95% confidence intervals.

### (c) Temporal activity

The PAMM results indicated a substantial, nonlinear effect of time on tiger and leopard visitation rates after human detection (tiger, e*df*  =  7.29, ref.*df*  =  8.28, *χ*^2^  =  273.50, *p* < 0.001; leopard, edf  =  1.22, ref.*df*  =  1.41, *χ*^2^  =  28.49, *p* < 0.001). Survey results showed that tiger detections were highest immediately after human presence, with the log hazard declining from 0.54 to about 0.14 within the first five days, reflecting a strong interaction signal (figure 4; supplementary material, figure S4). After the first 10 days, the log hazard for tiger occurrence progressively decreased over time until the 25th day (representing the interaction duration). Leopards exhibited a similar but weaker response to human activity. Specifically, the temporal dynamics of tiger event rates following the observation of humans were significantly different from those of leopards across our sites (95% CIs did not overlap; figure 4; supplementary material, figure S4).

**Figure 4.**
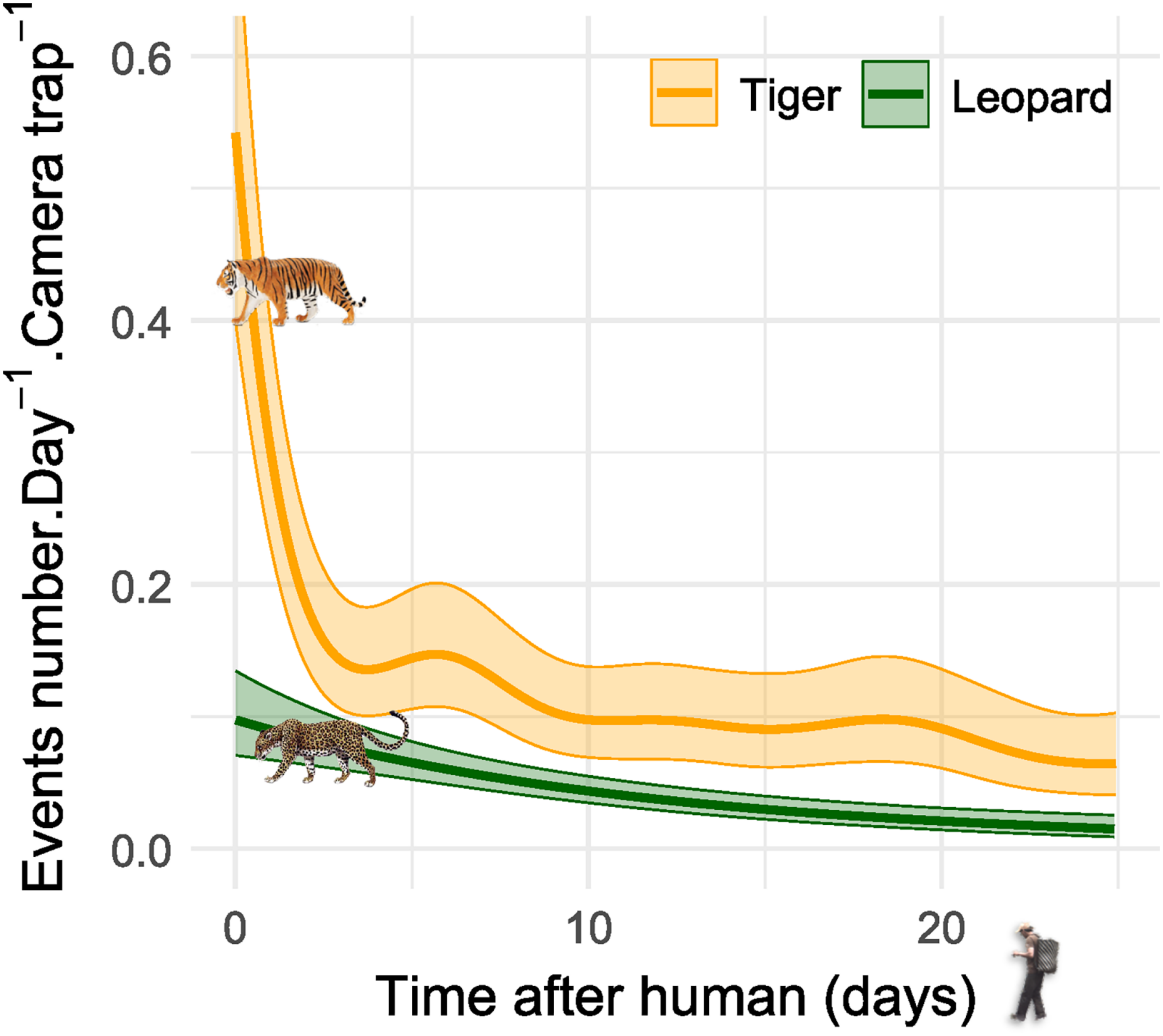
Dynamics of tiger and leopard visitation rates (log hazards, i.e., log-transformed daily visit events per camera trap) over time after a human presence, as predicted using piecewise exponential additive mixed models (PAMMs) and relative 95% confidence intervals.

When compared pairwise, the activity patterns of the leopards were significantly different from those of the tigers at both the low- and high-disturbance sites (all *p* < 0.01). In addition, leopards exhibited a distinct core activity range compared with tigers at lower or higher human disturbance sites (supplementary material, table S5). At the low-disturbance sites, the tigers’ core activity range was centred on dusk (from 14.55 to 21.05, in decimal hours; figure 5a), whereas the leopards moved to after dawn (from 05.81 to 10.73; figure 5c). At the high-disturbance sites, the tigers’ core activity range was mainly twilight and night (from 04.85 to 06.30 and from 16.92 to 21.66; figure 5b), whereas that of the leopards was adjusted to day (from 06.38 to 10.50 and from 14.09 to 18.22; figure 5d).

**Figure 5.**
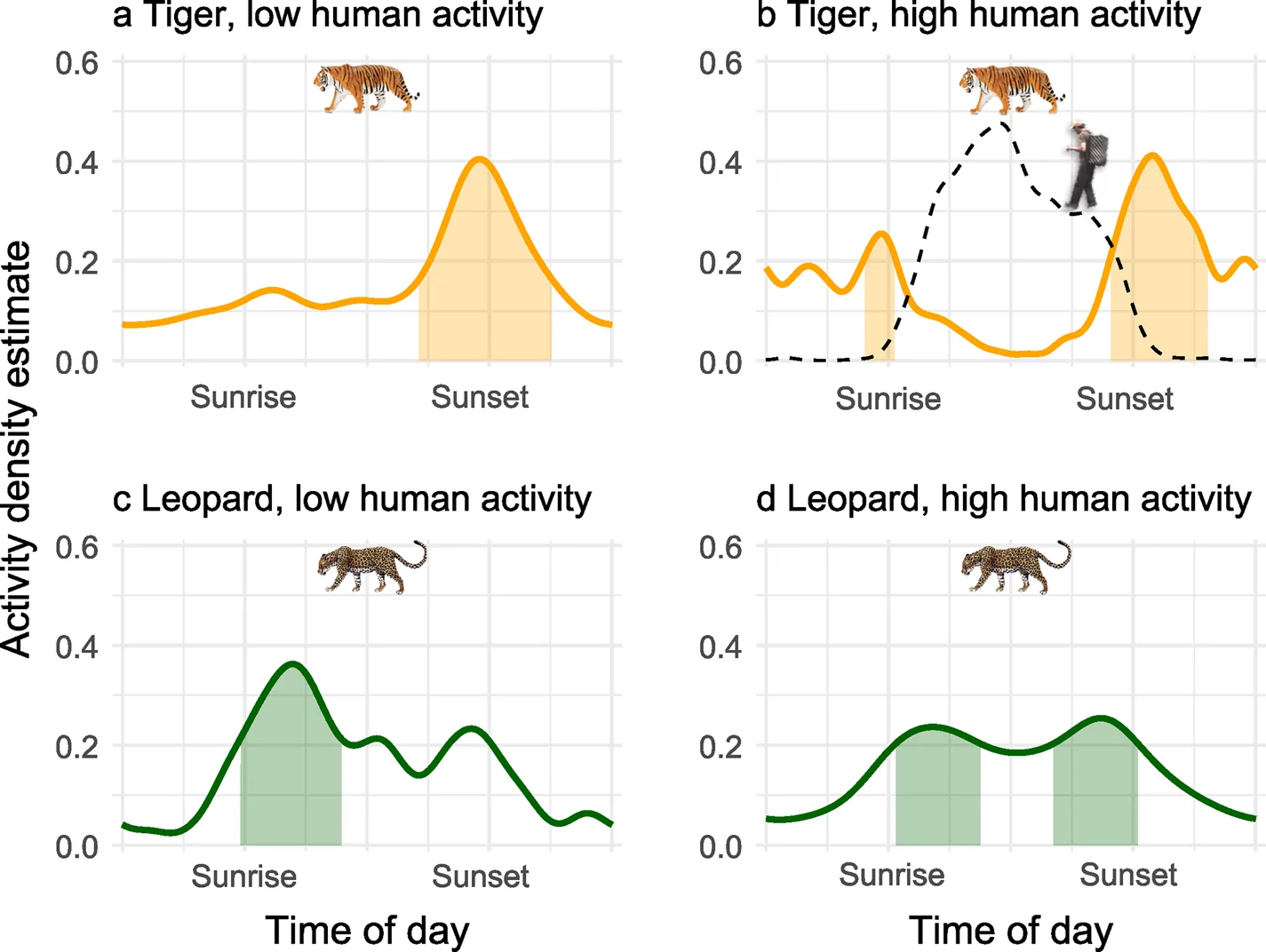
Kernel density estimates of the diel activity of tigers and leopards at (a,c) low and (b,d) high human activity sites using camera trap data collected in northeast China (2018–2019). The human relative abundance index (human RAI = 4.11) was taken for each species as the threshold to separate lower and higher rates of human activity. Lines represent mean estimates, and shaded areas represent activity range cores (50% isopleth). The dashed lines indicate the human activity patterns.

Across our sites, disturbance altered the diel overlap between the two predators. At low-disturbance sites (91 tiger detections and 322 leopard detections), the coefficient of overlap (Δ) between leopards and tigers was 0.67 (95% CI  =  0.57–0.77; supplementary material, figure S5), whereas at high-disturbance sites (880 tiger detections and 133 leopard detections), the Δ decreased to 0.56 (95% CI  =  0.47–0.64); however, both coefficients of overlap showed no significant difference (95% CIs overlapped).

Furthermore, disturbance altered the diel overlap between predator and prey. The MNLMM results revealed that human activities drove significant shifts away from diurnality in tigers (est. = 0.38, *p* < 0.001) and sika deer (Est. = 0.07, *p* < 0.01; figure 6a,c), but not in leopards (est. = 0.06, *p* = 0.41), roe deer (est. = 0.04, *p* = 0.22) and wild boar (Est. = 0.02, *p* = 0.37; figure 6b,d,e). Compared to those at the less disturbed sites, the temporal overlap between tiger and prey decreased significantly at the more disturbed sites, especially among roe deer and wild boar (95% CIs did not overlap; figure 7a).

**Figure 6.**
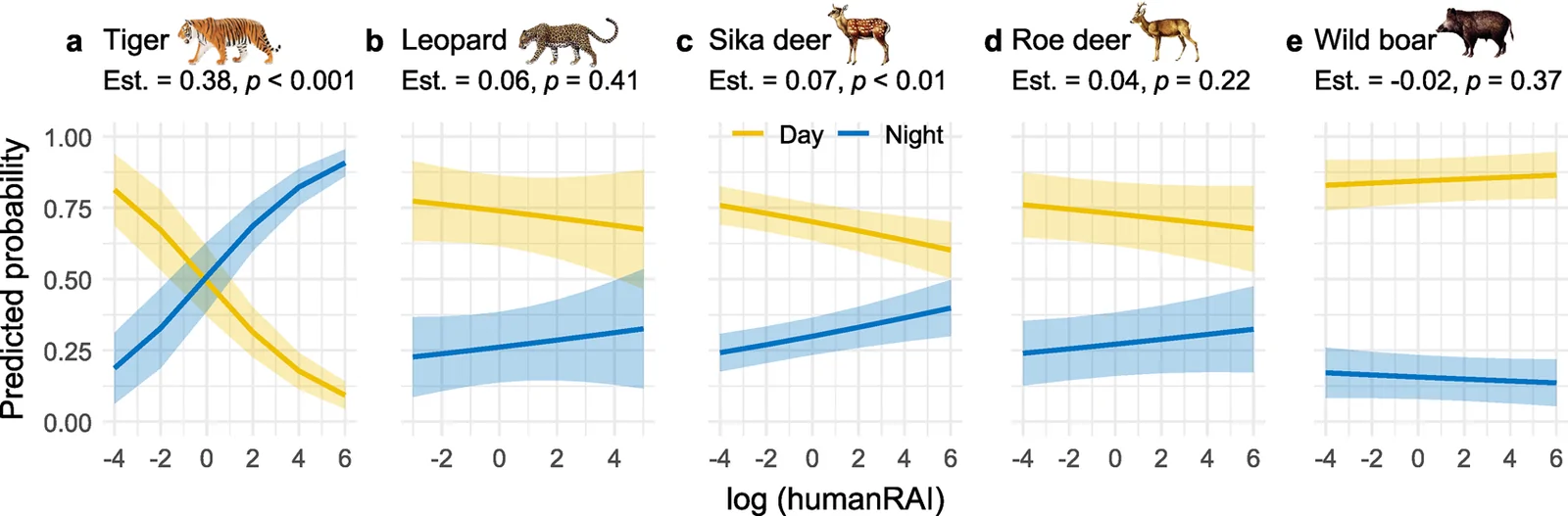
Effect of human activity on diel activity of (a) tigers, (b) leopards and (c–e) major prey species. Multinomial logistic mixed-effect models include human relative abundance (human RAI) as a fixed effect and camera site and month as random effects, with trend lines corresponding to the mean predicted probabilities of diel activity occurring during the day and night. Species-level diel activity responses (*p* < 0.05) show trends for night detections that have statistically significant slopes compared with day detections; the reference category and the shaded areas show the 95% confidence intervals.

**Figure 7.**
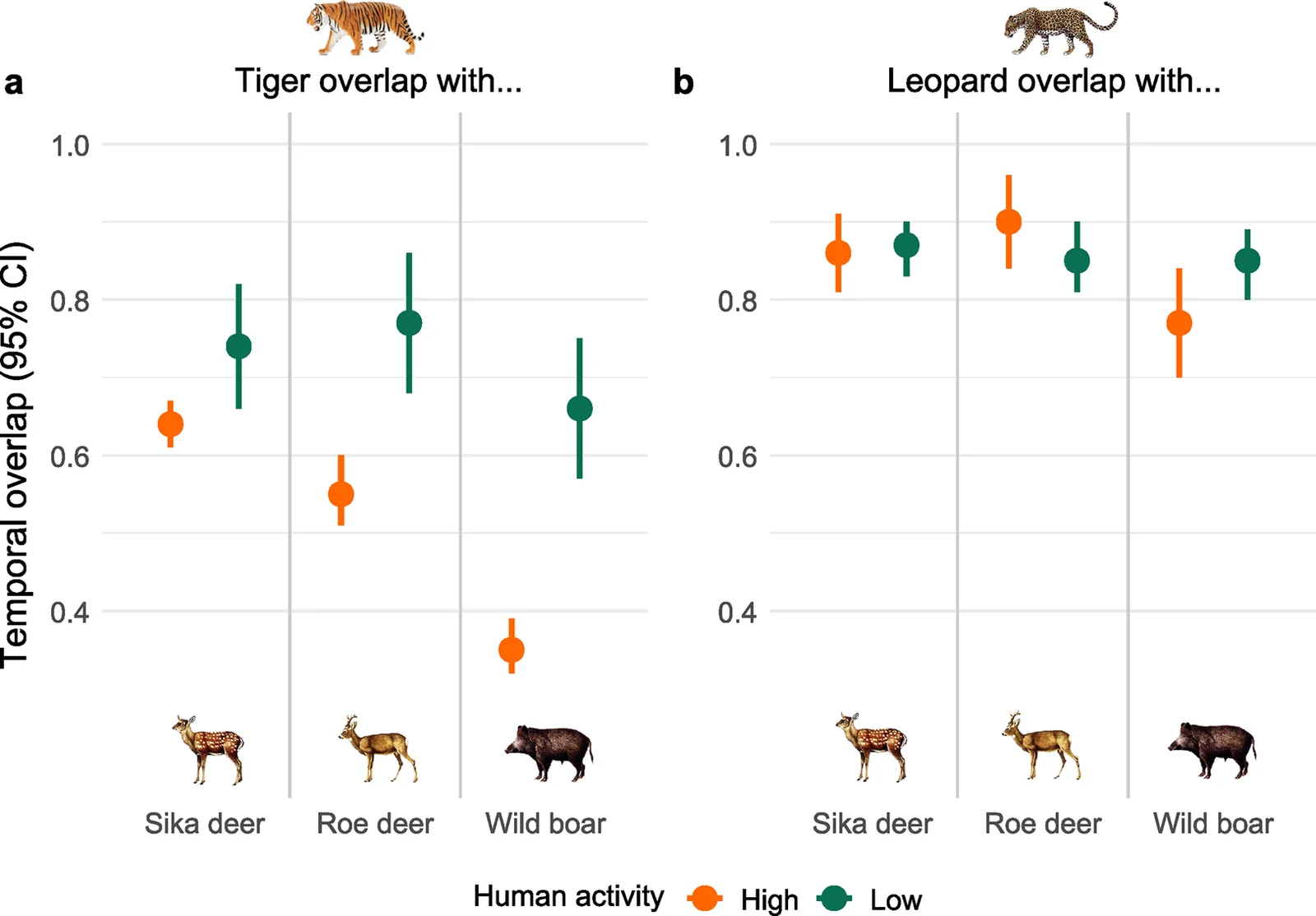
Changes in the overlap of the temporal niches of (a) tigers and (b) leopards with their major prey species between sites of low and high human activity. The coefficient of overlap ranges from 0, representing no overlap, to 1, representing total temporal overlap. The error bars show the bootstrapped 95% confidence intervals.

## 4. Discussion

The aim of this work was to assess how the presence of tigers and varying intensities of human activities influence the spatial and temporal dynamics of leopards on a short timescale (less than 1 year). Consistent with our predictions, leopard occupancy, colonization, and detection probabilities were significantly reduced in areas where tigers were present, highlighting the role of fear in structuring leopard spatial patterns. In addition, tigers displayed temporal shifts in activity in response to elevated human disturbance, likely reflecting behavioural adjustments to avoid human encounters. This also resulted in a noticeable decrease in diel overlap with ungulate prey. The leopards used temporal refuges, which may reduce encounters with tigers and human activity. We also found evidence that humans modify the direction and strength of interactions among carnivores [26]. We showed that co-occurrence between tigers and leopards was less frequent, except at lower human passage sites (human RAI ≤ 4.11), where habitat use by leopards was generally independent of tiger presence. This means that predictions from fear ecology theory can be extended from a predator‒prey system to predator, prey and human systems [16,47]. These findings suggest that direct human disturbance appears to coincide with changes in the spatial and temporal dynamics between these sympatric predators, with potential consequences for their coexistence in anthropogenically influenced habitat. Our results improve our understanding of how human activity at the site level affects the behaviour of large carnivores.

### (a) Tigers exerting top-down pressure on leopards

Leopards exhibited a lower likelihood of initial occupancy in areas where tigers were present, a trend that also extended to reduced colonization and detection probabilities. The marked declines in these metrics at tiger-occupied sites provide strong evidence for top-down regulation of leopard spatial dynamics by tigers. These findings indicate that tiger presence not only constrains leopard habitat use but also reduces the probability of successful colonization, thereby reinforcing the regulatory role of intraguild predation, as suggested earlier [63] and expected under the competition risk hypothesis [64]. Subordinate leopards either avoid landscapes where predation risk is greater and/or prey abundance is insufficient for larger, more dominant tigers to persist [18,37,65]; exploit landscapes where prey abundance is sufficient to support both subordinate and dominant carnivores [24,66]; or persist in areas where the tiger density falls below a certain threshold (at less than one adult tigers 100 km^−2^, a top-down regulatory effect by tigers on leopards diminishes) [20,67]. According to intraguild predation theory, the coexistence of ecologically similar carnivores may persist not only under conditions of low dominant predator abundance, which limits direct predation, but also via compensatory mechanisms such as access to alternative prey, behavioural adaptations by the subordinate species (e.g. increased vigilance), or adjustment of space use or activity timing to lower-risk places or periods while maintaining access to prey [68,69]. Our findings revealed significant reductions in leopard colonization at tiger-occupied sites, implying that leopards may exhibit spatial niche partitioning at a finer scale by preferentially occupying areas with reduced tiger densities [65].

### (b) Spatial interactions between tigers and leopards

We expected humans to shape the spatial relationship between tigers and leopards, and our results confirmed that variation in human use across sites moderated these interactions. In areas with relatively low human presence (human RAI ≤ 4.11), leopard occupancy was not affected by tiger presence, as the 95% CI of the estimated SIF included 1 according to the multispecies occupancy model. This suggests that in landscapes with no or few human disturbances, there is no evidence of interference competition. We suspect that the lower activity of tigers at these sites, temporal separation, and the availability of alternative prey together reduce the encounter rate, enabling leopards and tigers to coexist despite potential intraguild predation risk [8]. However, the low detection of tigers at less disturbed sites likely contributed to the uncertainty around this estimate (high 95% CI). Conversely, as the intensity of human activity surrounding sites increased, the SIF approached less than 1 (95% CI of the estimated SIF did not overlap with 1), suggesting that tigers and leopards co-occur less often than expected by chance.

Overall, these findings are consistent with the hypothesis that human presence alters tiger–leopard interactions by reducing their spatial overlap when perceived predation risk is elevated. Additionally, given that tigers use areas of high human use (see supplementary material, figure S3), leopards may actively avoid locations where humans and tigers co-create a landscape of fear, which may facilitate sympatry between locally competing carnivores at a relatively fine spatial scale but not large-scale spatial displacement [4,20]. In Chitwan National Park, Nepal, leopards tend to avoid areas used by tigers, but their ability to exploit human-modified environments allows them to survive in marginal habitats, leading to spatial separation between the two carnivores [65]. As human activities increasingly intrude into wildlife ranges worldwide, the behavioural adaptability of large carnivores may be crucial for coexistence. Such flexibility enables them to adjust activity patterns and habitat use in response to perceived human risk [40,44,49].

### (c) Temporal interactions between tigers and leopards

Our results partially support our hypothesis that tigers, but not leopards, significantly shifted their diel activity to nocturnal, which is likely to reduce predation risk in landscapes with greater diurnal human disturbance. Our data revealed that the RAI for tigers at high-disturbance sites (RAI = 2.97) was approximately 17 times greater than that at low-disturbance sites (RAI = 0.18). Congruent with previous research in our study system [41,70], tigers were frequently detected in the same areas used by pedestrians and vehicles, particularly along forest roads where their detection probability was higher. Our results of recurrent event analysis (i.e. spatiotemporal analyses) also confirmed this finding (figure 4). The increased detection of tigers near forest roads and smaller trails used by local people suggests they are energetically efficient means of traversing the landscape and feeding. Our results provide further evidence that tigers temporally avoid human activity without direct spatial avoidance, which supports evidence observed in Nepal [36,71].

Leopards are found to avoid humans in some parts of Asia [72], including in our study area [40]. Although human activity altered the activity patterns of tigers, when both tigers and leopards occur in areas regardless of the presence of humans, our data suggest that leopards temporally segregate their activity patterns (i.e. peaks and core activity range) to avoid tigers (figure 5), representing a major behaviour mechanism facilitating their coexistence in a cold temperate forest [20, 49]. However, diel overlap between tigers and leopards decreased more at more disturbed sites, supporting our hypothesis that leopards avoid competitive interactions by adjusting spatial and temporal activity when tigers are present. In parts of Asia with abundant prey and high densities of apex predators, leopards persist by adjusting their diet and using habitat flexibly at fine spatial scales, which enables them to avoid tigers locally while still sharing the broader landscape [4,21,24,67]. Our findings therefore highlight the importance of such behavioural plasticity in allowing subordinate carnivores to survive under pressures from both humans and dominant predators, and point to the value of conducting comparable studies in other human-impacted ecosystems.

### (d) Predator access to prey

This study demonstrates that human presence can exert non-consumptive effects on predator–prey dynamics, potentially shifting the temporal balance in favour of prey [31]. Our results suggested that tigers markedly decreased their temporal chances of encountering prey (in particular, the wild boars preferred by tigers) in response to diurnal human activity (Δ decreased from 0.66–0.77 to 0.35–0.64; see figure 7). At sites with higher levels of disturbance, however, leopards that remained largely diurnal still showed strong temporal overlap with their primary prey (Δ = 0.77–0.90), most likely because both predators and prey shifted their diel activity in similar ways, preserving the proportion of time they were active during the daily cycle [73]. These contrasting responses suggest that differences in how tigers and leopards adjust to human presence shape their interactions with ungulate prey. The leopard, as a subordinate species, exhibits relatively broader temporal tolerance and opportunistic ability, which represents an advantage in resource use and favours coexistence within the guild [74]. Besides, these further suggest that the temporal dimension may be especially important for subordinate leopards when balancing risks from humans and apex carnivores while maintaining access to prey [75]. Human presence may also potentially increase the nutritional benefits for tigers. For example, in areas with high human activity, tigers exhibited greater spatial overlap with their primary prey, sika deer (figure 3). Importantly, prey species are not directly threatened by humans in these parks, as hunting is prohibited. Therefore, in this study, a human presence appears to mediate the interaction between prey and predators both spatially and temporally while indirectly creating temporal refuges for prey that are more successful in avoiding tigers (but not leopards). This outcome aligns with the concept of a temporal human shield, whereby prey experience reduced predation risk during particular times of the day as a result of shifts in predator activity [31,76].

### (e) Conclusions and management implications

In summary, our results reveal how humans trigger complex species interactions across sympatric apex carnivores and how trophic position affects the behavioural responses and subsequent space and time use of dominant and/or subordinate carnivores across the landscape [26]. Understanding how species interact in a predator‒human landscape has significant implications for policy-makers and park managers in the context of managing the habitats of multiple predator and prey species or helping us identify factors that structure trophic-level interactions among carnivores [16,31]. Our findings also have broader implications for the conservation of leopards and tigers in complex, mixed-use landscapes in Asia. Given that human presence and activities, such as herding livestock, collecting forest products and travelling along forest roads and trails, are pervasive in the tiger conservation landscape in Asia [36,39], conservation efforts must consider the interplay among predators, prey and people. The incorporation of fine-scale spatiotemporal variation in response to human disturbance, which is considered a unique niche axis [16], into conservation plans can help managers inform guidelines for the effective management of nonexploitative human disturbance, a major global challenge.

In human–wildlife shared landscapes, management strategies should be context-specific. We therefore advocate limiting human activity at key times and sites of wildlife use to lessen disruptions to both predator–predator and predator–prey dynamics. Additionally, initiatives to include local communities in ecodevelopment or engaging local people in habitat management decisions may foster coexistence between wildlife and humans. Such measures could also ease conflicts between people and carnivores while advancing long-term conservation objectives for both the species and the ecosystems they inhabit [36,77].

## Supplementary Material



## Data Availability

The data supporting this study are publicly available from Zenodo [78]. Supplementary material is available online [79].
