# Supplementary material for: Fear of humans mediates spatiotemporal interactions between endangered large Asian felids
Source: Proc Biol Sci. 2026 Oct 7;293(2080):20261232. doi: 10.1098/rspb.2026.1232 (PMC13641897; doi:10.1098/rspb.2026.1232)
Supplement: Supplementary file 1 [file rspb.2026.1232.zip › rspb.2026.1232/rspb20261232_si_001.docx]

**Electronic Supplementary Material**

for the article published in Proceedings of the Royal Society B

(https://doi.org/10.1098/rspb.2026.1232)

**Fear of humans mediates spatiotemporal interactions between endangered large Asian felids**

Jiawei Feng ^1, 2^, Dawei Wang ^1, 2*^, Zhilin Li ^3^, Xintong Wei^1, 2^, Jianping Ge ^1, 2^ Tianming Wang ^1, 2*^

^1^ Ministry of Education Key Laboratory for Biodiversity Science and Ecological Engineering, College of Life Sciences, Beijing Normal University, Beijing, 100875, China

^2^ National Forestry and Grassland Administration Key Laboratory for Conservation Ecology of Northeast Tiger and Leopard, Beijing, 100875, China

^3^ College of Life Sciences, Tianjin Normal University, Tianjin, 300387, China

* Corresponding authors:

Dawei Wang, e-mail: daweiwang@bnu.edu.cn

Tianming Wang, e-mail: wangtianming@bnu.edu.cn

**Table S1.** Summary of independent detections of each species across all 329 camera-trap sites monitored from January 2018 to September 2019, including detections at sites with lower and higher human activity. The human relative abundance index (human RAI = 4.11, see *Methods*) was taken for each species as the threshold to separate lower and higher rates of human activities.

| **Species/human** | **Total detections** | **Detections at less disturbed sites** | **Detections at more disturbed sites** |
| --- | --- | --- | --- |
| Tiger (*Panthera tigris*) | 971 | 91 | 880 |
| Leopard (*Panthera pardus*) | 455 | 322 | 133 |
| Sika deer (*Cervus nippon*) | 15,118 | 9,207 | 5,911 |
| Roe deer (*Capreolus pygargus*) | 2083 | 1,620 | 463 |
| Wild boar (*Sus scrofa*) | 3,383 | 2,733 | 650 |
| Human (including vehicles) | 7,160 | 201 | 6,959 |

**Table S2.** Parameter summaries from a dynamic occupancy model that assumes that the site occupancy of a leopard (*Panthera pardus*) varies due to the presence of a tiger (*P. tigris*). “Tiger” refers to all sites where tigers were observed, “Leopard without tiger” refers to sites where leopards were observed but tigers were not, and “Leopard with tiger” refers to sites where leopards and tigers were observed at the same sites.

| **Species** | **Median** | **95% CI** |
| --- | --- | --- |
| **Tiger** | | |
| Detection | 0.019 | 0.014–0.026 |
| Occupancy | 0.239 | 0.134–0.388 |
| Colonization | 0.133 | 0.096–0.185 |
| Persistence | 0.480 | 0.277–0.682 |
| **Leopard without tiger** | | |
| Detection | 0.015 | 0.011–0.019 |
| Occupancy | 0.429 | 0.297–0.581 |
| Colonization | 0.055 | 0.019–0.116 |
| Persistence | 0.900 | 0.758–0.974 |
| **Leopard with tiger** | | |
| Detection | 0.001 | 0.000–0.002 |
| Occupancy | 0.024 | 0.013–0.044 |
| Colonization | 0.004 | 0.000–0.014 |
| Persistence | 0.969 | 0.887–0.998 |

**Table S3.** Metadata table for parameters used to estimate detection, occupancy, colonization, and persistence rates between leopards and tigers within a dynamic occupancy model. Effective sample size is the calculation of the equivalent number of independent samples that would contain the same posterior accuracy as the correlated samples from a Markov chain Monte Carlo. Model convergence was assessed by visually inspecting trace plots and ensuring that Gelman-Rubin diagnostics were < 1.1 (all parameters were < 1.1). We assessed presence/absence of leopards and tigers using camera traps at sites distributed across Northeast China.

| **Parameter** | **Scale** | **Description** |
| --- | --- | --- |
| a[x,y,z] | logit | Species by Parameter by Process array for the influence of tiger on leopard colonization and persistence. For the first index, the 2 represents leopard. For the second index, a 1 represents the average log-odds difference tiger presence has on the given process and a 2 represents the log-odds difference tiger presence has on leopard’ response to human activity. For the third index, a 1 represents colonization while a 2 represents persistence. |
| da[x,y] | logit | Species by Parameter array for the influence of tiger on leopard detectability. For the first index, 2 = leopard. For the second index, the 1 represents the average log-odds difference tiger presence has on leopard detectability while the 2 represents the log-odds difference tiger presence has on leopards’ response to human activity. |
| psi0[i] | logit | Initial Occupancy intercept [species]. 1 = tiger. 2 = leopard. |
| psi_human[i] | logit | Initial Occupancy Slope Term [species] 1 = tiger. 2 = leopard. |
| pmu[i] | logit | Persistence Intercept [species]. 1 = tiger. 2 = leopard. |
| lp[i] | logit | Detection Intercept [species]. 1 = tiger. 2 = leopard. |
| gmu[i] | logit | Colonization Intercept [species]. 1 = tiger. 2 = leopard. |
| gb[i] | logit | Colonization Slope Term [species]. 1 = tiger. 2 = leopard. |
| pb[i] | logit | Persistence Slope Term [species]. 1 = tiger. 2 = leopard. |
| lpb[i] | logit | Detection Slope Term [species]. 1 = tiger. 2 = leopard. |

| **Parameter** | **Species** | **Process** | **Covariate** | **Median** | **95%CI** | **ESS** |
| --- | --- | --- | --- | --- | --- | --- |
| γ (a[2,1,1]) | Leopard | Colonization | Human (tiger influence) | -2.58 | -5.74 – -0.98 | 1231 |
| ε (a[2,2,1]) | Leopard | Persistence | Human (tiger influence) | -0.26 | -1.35 – 0.80 | 5709 |
| γ (a[2,1,2]) | Leopard | Colonization | Human (leopard only) | 1.11 | -0.20 – 3.12 | 1925 |
| ε (a[2,2,2]) | Leopard | Persistence | Human (leopard only) | -0.78 | -2.58 – 0.30 | 1999 |
| p (da[2,1]) | Leopard | Detection | Human (tiger influence) | -3.42 | -3.78 – -3.08 | 9369 |
| p (da[2,2]) | Leopard | Detection | Human (leopard only) | -0.58 | -0.96 – -0.20 | 4834 |
| ψ (psi0[1]) | Tiger | Initial Occupancy | Human | -1.16 | -1.87 – -0.45 | 8138 |
| ψ (psi0[2]) | Leopard | Initial Occupancy | Human | -0.29 | -0.86 – 0.33 | 3612 |
| ψ (psi_human[1]) | Tiger | Initial Occupancy | Human | 1.31 | 0.66 – 1.98 | 9620 |
| ψ (psi_human[2]) | Leopard | Initial Occupancy | Human | 0.39 | -0.3 – 1.44 | 1946 |
| ε (pmu[1]) | Tiger | Persistence | Human | -0.08 | -0.96 – 0.76 | 2340 |
| ε (pmu[2]) | Leopard | Persistence | Human | 2.20 | 1.14 – 3.62 | 1474 |
| p (lp[1]) | Tiger | Detection | Human | -3.93 | -4.26 – -3.62 | 3110 |
| p (lp[2]) | Leopard | Detection | Human | -4.19 | -4.47 – -3.94 | 3846 |
| γ (gmu[1]) | Tiger | Colonization | Human | -1.87 | -2.24 – -1.48 | 5255 |
| γ (gmu[2]) | Leopard | Colonization | Human | -2.85 | -3.97 – -2.03 | 2544 |
| β (gb[1]) | Tiger | Colonization | Human | 0.34 | 0.00 – 0.67 | 7641 |
| β (gb[2]) | Leopard | Colonization | Human | 0.27 | -0.43 – 1.03 | 6287 |
| β (pb[1]) | Tiger | Persistence | Human | 1.06 | 0.38 – 1.74 | 2487 |
| p (lpb[1]) | Tiger | Detection | Human | 0.60 | 0.39 – 0.81 | 3148 |
| β (pb[2]) | Leopard | Persistence | Human | 0.32 | -0.70 – 1.67 | 2853 |
| p (lpb[2]) | Leopard | Detection | Human | 0.18 | -0.12 – 0.48 | 3457 |

**Table S4.** Parameters used to estimate detection (**p**), occupancy (**ψ**), colonization (**γ**), and persistence (**ε**) rates between leopards (*Panthera pardus*) and tigers (*P. tigris*) within a dynamic occupancy model. We included one human activity covariate at each camera site for all logit-linear predictors (i.e., initial site occupancy, colonization, persistence, and detection). Effective sample size (**ESS**) is the calculation of the equivalent number of independent samples that would contain the same posterior accuracy as the correlated samples from a Markov chain Monte Carlo. We assessed presence/absence of leopards and tigers using camera traps at sites distributed across Northeast China. See supplemental Table S1 for a description of each parameter and what each index represents.

**Table S5.** Activity range conditioned to kernel isopleth 50% (activity range core, in decimal hours) for tigers and leopards across higher and lower human activity sites in Northeast China (2018-2019).

| Species | Intervals | Range |
| --- | --- | --- |
| Low human activity | | |
| Tiger | 14.55 – 21.05 | 6.50 |
| Leopard | 05.81 – 10.73 | 4.92 |
|  |  |  |
| High human activity | | |
| Tiger | 04.85 – 06.30 | 1.45 |
|  | 16.92 – 21.66 | 4.74 |
| Leopard | 06.38 – 10.50 | 4.12 |
|  | 14.09 – 18.22 | 4.13 |

**Figure S1.** Map of the study area in the eastern part of Northeast Tiger and Leopard National Park (NTLNP) showing the 329 camera trapping stations (2018-2019). The insert shows the location of the study area along the China–Russia border. The right panel shows camera traps deployed in the field. Photo credit: T. Wang.

**Figure S2.** Species interaction factor (SIF) of leopards and tigers by month in relation to human activity in Northeast China. An SIF of 1 indicates expected spatial overlap, whereas an SIF > 1 indicates that the species interact more often than expected, and an SIF < 1 indicates that the species interact less often than expected. The shaded areas show the 95% confidence intervals. The vertical dotted line represents log[humanRAI] = 1.44.

**Figure S3.** Occupancy probability of leopards and tigers (a, b), as well as the effect tiger presence on leopard occupancy (c, d), in relation to human activity in Northeast China. The shaded areas show the 95% confidence intervals.

**Figure S4.** Recurrent event surveys and visitation-rate dynamics of Amur tigers and Amur leopards following human detections. Recurrent-event surveys showing the distribution of Amur tiger observations following human detections (a) and Amur leopard observations following human detections (c). Green points represent recurrent events, and grey horizontal lines represent individual surveys. Piecewise exponential additive mixed model (PAMM) predictions show the dynamics of tiger visitation rates over time following human detections (b) and leopard visitation rates over time following human detections (d). Black lines represent the predicted visitation rates, and grey shaded areas indicate the corresponding 95% confidence intervals.

**Figure S5.** Kernel density function plots and overlap estimation of tigers and leopards in low and high human activity sites using the camera-trap data collected in Northeast China (2018-2019). The human relative abundance index (human RAI = 4.11) was taken in each species as the threshold to separate lower and higher rates of human activities.
